# Citrus peel extract–enriched gelatin edible films as natural preservatives to enhance the shelf life and quality of chicken burgers

**DOI:** 10.1007/s44463-026-00110-8

**Published:** 2026-09-28

**Authors:** Farhat Khalily, İlknur Uçak, Qurat ul ain Sajid, Muhammad Umair Asghar

**Affiliations:** 1 Department of Animal Production and Technologies, Faculty of Agricultural Sciences and Technologies, Niğde Ömer Halisdemir University, Niğde, Turkey; 2 Department of Animal Nutrition and Feed Sciences, Wrocław University of Environmental and Life Sciences, 25 C.K. Norwida St., 51-630, Wrocław, Poland

**Keywords:** Active packaging, Citrus peel extract, Shelf life, Gelatin edible film, Chicken burgers, Antimicrobial, Frozen storage, Lipid oxidation, Natural preservative

## Abstract

This study examines the effects of edible gelatin films enriched with citrus fruit peel extracts (grapefruit, orange, and lemon) at concentrations of 1%, 2%, and 4% on the quality and shelf life of chicken burgers during frozen storage at − 18 °C, over 147 days. Significantly, the duration of shelf life was increased by inhibiting two factors, microbial and oxidation, in chicken burgers by using enriched gelatin films. On day 147, 4% grapefruit peel extract showed significantly lower peroxide value (4.50 meq O₂/kg), thiobarbituric acid value (0.74 mg MDA/kg), total volatile basic nitrogen (11.20 mg N/100 g), and pH (6.77) than the unwrapped control samples (*p* ≤ 0.05). Moreover, microbial analysis revealed that 4% citrus fruit peel extracts significantly inhibited the burgers’ total aerobic psychrophilic count, total mesophilic bacteria, total coliform bacteria, total lactic acid bacteria, total yeast and mold count compared with the control and other groups. Grapefruit peel exhibited the highest antioxidant and phenolic content, followed by orange and lemon peels. Overall, gelatin films fortified with citrus peel extracts 4% showed improved an-tioxidant and antibacterial capabilities, indicating their potential as natural active packaging materials for meat preservation.

## Introduction

Chicken meat is one of the most widely consumed meats worldwide and serves as an important source of animal protein (Asghar et al., 2021; Gumowski et al., 2025). It is considered a healthier dietary option due to its high nutritional content, including proteins, low fat, free amino acids, vitamins, and minerals (Sierżant et al., 2023; Konkol et al., 2025). However, chicken meat is highly perishable, even when stored at low temperatures (Katiyo et al., 2020). Key factors contributing to spoilage include oxygen, natural enzymes, and microbial activity (Asghar et al., 2022; Naimati et al., 2022). During refrigerated storage, the metabolic activities of psychrotrophic bacteria such as Pseudomonas spp., Enterobacteriaceae, Brochothrix thermosphacta, and Lactic acid bacteria often lead to physicochemical changes (Naimati et al., 2025; Casaburi et al., 2015). Generally, these spoilage processes in chicken meat cause changes in pH, slime formation, and degradation of structural components (Tura et al., 2024). Therefore, presently, researchers are researching naturally derived techniques to inhibit microbial growth and oxidation, including the use of biopolymers (gelatin, chitosan, alginate, starch, agar, carrageenan, collagen, and keratin) as preservation agents (Sarfraz et al., 2024). These biopolymers are transformed into bioplastics, edible films, or functional additives.

Edible film is a thin layer of edible material applied directly to the surface of the food product. Its primary function is to reduce spoilage factors such as microbial growth, oxygen and water vapor permeability, and the loss of flavor volatiles, thereby extending the shelf life of food products (Kong et al., 2023). These films are commonly produced by using biopolymers such as polysaccharides and proteins (Mahmoud et al., 2017). Therefore, the physical properties and structural characteristics of these biopolymers can vary depending on the composition and matrix structure (Rangaraj et al., 2021). Gelatin is one of the most significant and remarkable biopolymers. It is a protein derivative, is widely used in food applications due to its unique properties, including its ability to form gels, surface activity, viscosity control, and excellent film-forming capacity (Khedri et al., 2021). However, despite these advantages, pure gelatin and gelatin-based films are limited in application due to their lack of inherent antioxidant and antimicrobial properties (Rangaraj et al., 2021). To overcome this limitation, recent research has focused on enhancing the functional properties of biodegradable films by incorporating natural plant-derived antioxidants, such as extracts and essential oils (Li et al., 2014). These plant extracts, rich in phenolic compounds and derived from various plant parts (e.g., fruits, leaves, seeds, and peels), have been used effectively as active ingredients in the development of gelatin-based films and coatings (Li et al., 2014). For this reason, citrus fruits are among the most popular fruit crops worldwide due to their attractive colors, delicious taste, and pleasant aroma (Yun & Liu, 2022). Global citrus production has in-creased by more than 157 million tons per year. These fruits belong to the Rutaceae family, which includes more than 1300 species (Kumar et al., 2022). Orange, lemon, mandarin, lime, and grapefruit are some of the most commonly consumed citrus fruits globally (Lu et al., 2018). However, approximately 50–60% of citrus fruit waste becomes inedible after consumption or processing, including peels, seeds, pulp, and segment residues (Teigiserova el at., 2021).

Many studies have explored the potential applications of citrus peel in the food industry because they are rich source of valuable bioactive components, including essential oils, vitamins, flavonoids, dietary fiber, proteins, pectin, and various phenolic compounds (Wedamulla et al., 2022). The antioxidant and antimicrobial properties of citrus peel extracts are attributed to the presence of bioactive compounds, including phenolic acids, essential oils, and flavonoids (e.g., hesperidin and naringin), which have demonstrated considerable potential as natural preservatives in food systems, as described in prior studies (Lu et al., 2018; Singh et al., 2020). Previous studies have shown that incorporating citrus peel-derived bioactive compounds into biopolymer matrices can modify important physical properties, including water vapor permeability, oxygen barrier capacity, and mechanical strength (Yun & Liu., 2022; Moura-Alves et al., 2023). These modifications can significantly enhance the antioxidant and antimicrobial properties of edible films, thereby improving food quality and extending shelf life (Yun & Liu, 2022; Moura-Alves et al., 2023). In addition, citrus peel extracts are rich in phenolic compounds, flavonoids, and other phytochemicals that have demonstrated considerable potential as natural preservatives in food systems (Jridi et al., 2019; Wedamulla et al., 2022). However, despite these promising findings, limited information is available regarding the comparative effectiveness of grapefruit, orange, and lemon peel extracts incorporated into gelatin-based edible films for preserving chicken burgers during prolonged frozen storage. To the best of our knowledge, few studies have systematically compared the preservative performance of these citrus peel extracts at different incorporation levels under identical formulation and storage conditions while simultaneously evaluating their effects on physicochemical characteristics, oxidative stability, and microbiological quality. Therefore, the present study aimed to evaluate the effects of gelatin edible films enriched with 1%, 2%, and 4% grapefruit, orange, and lemon peel extracts on the quality and shelf life of chicken burgers during 147 days of frozen storage at − 18 °C. Unlike previous studies, which primarily investigated individual citrus peel extracts or edible films separately, the present study provides a systematic comparative assessment of grapefruit, orange, and lemon peel extracts incorporated into gelatin-based edible films under identical formulation and frozen-storage conditions. The findings contribute to the development of sustainable active packaging systems by demonstrating the potential of citrus processing by-products as natural preservatives for meat products while supporting the valorization of agricultural waste.

## Materials and methods

### Preparation of citrus peel extracts

Preparation of citrus peel extracts. In this study, grapefruit, orange, and lemon were obtained from a local market in Niğde. Then the fruits were transferred to the laboratory, where they were washed with tap water. After washing, the fruits were peeled. Furthermore, the grapefruit, orange, and lemon peels were dried separately in the laboratory convection oven at 45 °C for 48 h. Then, the ground peel powder was used for extraction. For extraction, 10 g of peel powder were added to 100 mL of 70% ethanol to make a mixture, and the mixture was subjected to ultrasonic treatment (CUB-5, Citizen, 40 kHz, 220–240 V) at 30 °C for 45 min. The mixtures were then filtered through filter paper to separate the ethanol, which was used in a rotary vacuum evaporator at 45 °C, and after 45 min, the extract was obtained.

### Chicken burger preparation

Approximately 22 kg of fresh chicken meat (breast and leg portions) was purchased from Bayaz Market, transported with ice, and then cleaned, washed, and deboned. Chicken burgers were prepared using minced chicken breast (69.5%), wheat flour (10.0%), corn starch (10.0%), soybean oil (5.0%), rice flour (2.0%), salt (1.0%), onion powder (2.0%), garlic powder (0.20%), black pepper (0.10%), thyme (0.10%), and cumin (0.10%). Then, a 100 g of burger patties (1 × 8 cm) were shaped and individually wrapped between two films. To facilitate comparisons, samples were divided into 11 groups: a control (untreated); a gelatin film group without extract (C-G); three groups with gelatin films enriched with grapefruit extract at 1%, 2%, and 4% (G-G1, G-G2, G-G4); three groups with gelatin films enriched with orange extract at 1%, 2%, and 4% (G-O1, G-O2, G-O4); and three with lemon extract at 1%, 2%, and 4% (G-L1, G-L2, G-L4). Chicken burger samples were stored at − 18 °C for 147 days, a storage period selected to simulate the extended frozen shelf life commonly applied to commercial frozen chicken burger products. Physicochemical and microbiological analyses were conducted on Days 0, 21, 42, 63, 84, 105, and 147. These sampling intervals were chosen to capture both the initial changes and the progressive physicochemical and microbiological alterations occurring throughout frozen storage, thereby enabling a comprehensive evaluation of product quality and storage stability over time.

### Preparation of gelatin film-forming solutions

Gelatin films were prepared in 3 steps by using the casting technique described by Gómez-Estaca et al. (2009). In this study gelatin was used as a film-forming agent. Glycerol was added to increase flexibility, and d-sorbitol was used as a plasticizer. Furthermore, citrus extracts were into added into the gelatin solution as a natural functional additives. The concentrations of 1%, 2%, and 4% citrus peel extracts were selected based on previously published studies reporting effective incorporation levels in edible film formulations while maintaining satisfactory film-forming characteristics, as well as the experimental design established for the present investigation. These concentrations were considered representative of low, intermediate, and high incorporation levels for evaluating their preservative potential. A 3% concentration was not included because it was not part of the original experimental design.

### Physical and chemical analysis

#### Determination of sample pH

The pH was determined according to the method described by Manthey et al. (1988). A digital pH meter (Hanna HI2211, Hanna Instruments, Romania) was used to measure the pH of chicken burger samples. An approximately 10-gram sample was mixed with 90 mL of distilled water in a laboratory homogenizer for 1–2 min. The pH meter was calibrated with standard buffer solutions of pH 4.0 and 7.0 before measurement. After homogenizing the sample, the electrode was placed and the pH value was recorded once the reading stabilized. The measurements were taken three times, and the average values were used to represent the results.

#### Total volatile basic nitrogen (TVB-N)

The total volatile basic nitrogen (TVB-N) content was measured according to the method described by Schormüller (1968). In brief, 20 g of homogenized chicken burger were subjected to 50 ml of distilled water. Then, approximately 1 spatula (or spoon) of magnesium oxide (MgO) was added as an antifoaming agent. The distillate was collected in a receiving flask containing 0.1 N hydrochloric acid (HCl), where the volatile bases were absorbed. Subsequently, the excess acid was back-titrated using an equal normality base solution (0.1 N NaOH). The volume of titrant used was recorded, and the amount of TVB-N was calculated and expressed as milligrams of nitrogen per 100 g of sample (mg/100 g).

#### Peroxide value (PV)

To determine the peroxide value (POV) of chicken meat, it was determined according to the method described by AOAC (1990) and AOAC (1998). The chemical used to determine the peroxide value was as follows: 1 g sample, 30 mL of a chloroform–acetic acid solution (2:3, v/v), 1 mL of a saturated potassium iodide (KI), 75 mL of distilled water, 0.5 mL of starch, and 0.1 M sodium thiosulfate (Na₂S₂O₃). The peroxide values of the samples were calculated according to the expressed in meq/kg.

#### Thiobarbituric acid reactive substances (TBARS)

Thiobarbituric acid reactive substances (TBARS) were determined following the method described by AOAC (1998). For 0.100 g of the sample, the following chemicals were used in the analysis: 5 mL of n-butanol, 5 mL of thiobarbituric acid (TBA) reagent. The absorbance of the resulting supernatant was measured at 530 nm using a spectrophotometer. The results were calculated with the formula given below and expressed as mg malondialdehyde / kg sample.

#### Microbiological analysis

Microbiological counts were performed by homogenizing a 10 g sample in 90 mL of Ringer’s solution using a stomacher. Psychrophilic bacteria were counted on Tryptone Glucose Yeast (TGYE Agar) after incubation at 7–10 °C for one week. Mesophilic bacterial counts were determined by the pour plate method using Tryptone Glucose Yeast (TGYE Agar) medium, with incubation at 30 °C for 2 days (Gelman & Benjamin, 1989). Coliform bacteria were counted on Violet Red Bile Agar (VRBA) medium incubated at 37 °C for 24–48 h. Lactic acid bacteria were counted on Man Rogosa and Sharpe (MRS) agar, which supports their growth, incubated at 30 °C for 48 h. Molds and yeasts were counted on BAM Media M127 after incubation at 25 °C for 5 days (AOAC, 1998). All microbial counts were expressed as log CFU/g.

#### Bioactive component analysis

##### Antioxidant activity

The antioxidant activity of grapefruit, orange, and lemon peel extract was determined according to the method described by Re et al. (1999). The antioxidant activity of the samples was calculated using the strong linear correlation (*r* = 0.971) ABTS assay and showed as (µmol TE/g).

##### Total phenolic content

The Folin-Ciocalteu colorimetric technique was used for the resolution of total phenolic contents (TPC) (Spanos & Wrolstad, 1990). Total phenolic contents of the samples were calculated using the strong curve (*r* = 0.995) and the outcome was expressed as mg gallic acid equivalents per gram (mg GAE/g).

### Statistical analysis

Each citrus peel extract was prepared in three independent batches, and each gelatin film formulation was fabricated in three independent replicates. All physicochemical and microbiological analyses were performed in triplicate (*n* = 3) for each treatment at each sampling interval. The results are expressed as mean ± standard deviation (SD). Statistical analysis was performed using IBM SPSS Statistics (IBM Corp., Armonk, NY, USA). Differences among treatment means were evaluated using one-way analysis of variance (ANOVA), followed by Tukey’s multiple comparison test. Statistical significance was considered at *p* < 0.05.

## Results and discussion

### Physical and chemical properties of gelatin films

Citrus peel extracts had a major impact on the gelatin films’ mechanical qualities, improving their flexibility and usefulness. Two important factors that determine the freshness of chicken meat are pH and volatile basic nitrogen (TVB N) values, and an increase in these two values indicates deterioration in the meat. Initially, the pH value of all samples was 6.10. Over the 147-day storage period, a slight increase in pH was noted in all groups (Table 1). Chicken burgers treated with gelatin films containing higher concentrations (4%) of citrus peel extracts showed a small elevation in pH, indicating a smooth biochemical reaction. At the end of storage, Groups G-G4, G-O4, and G-L4 were noted at 6.77, 6.88, and 6.80, respectively. In contrast, Groups C and G-C exhibited significantly higher pH values of 7.13. The progressive increase in pH observed during frozen storage is more appropriately associated with the accumulation of alkaline metabolites, such as ammonia and biogenic amines, produced during spoilage-associated microbial metabolism and protein decomposition rather than protein degradation alone. This interpretation is further supported by the concurrent increase in TVB-N values observed throughout the storage period, indicating the gradual formation of volatile nitrogenous compounds associated with quality deterioration. Similar observations were reported by Farokhzad et al. (2023), who attributed the higher pH values observed in untreated control samples to more extensive spoilage-related biochemical changes during storage. Furthermore, increasing the concentration of grapefruit, orange, and lemon peel extracts incorporated into the gelatin edible films effectively slowed the increase in pH during frozen storage. This protective effect may be attributed to the antioxidant properties and intrinsic antimicrobial activity of citrus peel phytochemicals, including phenolic compounds and flavonoids, which help reduce spoilage-associated biochemical reactions and maintain the physicochemical stability of chicken burgers during storage (Ebrahimian et al., 2023).

Similarly, as shown in Table 1, the control sample exhibited higher TVB-N values compared with the treatment samples, indicating greater spoilage activity and protein degradation in the meat (Manthey et al., 1988). However, films with extracts (G-G4, G-O4, G-L4), (G-G2, G-O2, G-L4), and (G-G1, G-O1, G-L1) had a significant effect on the TVB-N value of the burgers during frozen storage (*P* < 0.05). At the end of storage, the lowest TVB-N value, 11.20 (mg N/100 g), was observed in the gelatin-coated samples containing grapefruit peel with 4% extract (G-G4). While the highest value was noted in the control group 20.30 (mg N/100 g). As shown by the Egyptian Organization for Standardization and Quality Control (EOSQC, 2005), the freshness of chicken meat is 10–15 (mg N/100 g), and the rejection level is 20 (mg N/100 g).

As illustrated in Table 1, the pH and TVB-N values of all samples increased progressively during storage. No significant differences in pH or TVB-N values were observed among the treatment groups at Day 0. The initial measurements were performed immediately after the application of the gelatin edible films and packaging, prior to the occurrence of storage-related physicochemical and microbiological changes. Consequently, all samples exhibited comparable initial quality characteristics, whereas treatment-related differences became evident only during subsequent frozen storage. Compared with the control, chicken burgers coated with grapefruit, orange, and lemon peel extract-incorporated gelatin films consistently exhibited lower pH and TVB-N values throughout frozen storage, indicating improved preservation of physicochemical quality. These findings suggests that the incorporation of citrus peel extracts into the gelatin films contributed to maintaining product quality during frozen storage through their antioxidant properties and intrinsic antimicrobial potential, thereby reducing spoilage-associated biochemical changes.

**Table 1:** Change in pH and TVB-N values of chicken burgers coated with gelatin films enriched with grapefruit, orange, and lemon peel extracts during frozen storage (− 18 °C) for 147 days

Groups											
Storage (days)	C	C-G	G-G1	G-G2	G-G4	G-O1	G-O2	G-O4	G-L1	G-L2	G-L4
PH											
0	6.43 ± 0.06^Aef^	6.43 ± 0.06^Ac^	6.43 ± 0.06^Ac^	6.43 ± 0.06^Ac^	6.43 ± 0.06^Ac^	6.43 ± 0.06^Ae^	6.43 ± 0.06^Ac^	6.43 ± 0.06^Ac^	6.43 ± 0.06^Acd^	6.43 ± 0.06^Ac^	6.43 ± 0.06^Ab^
21	6.27 ± 0.12^Bef^	6.30 ± 0.10^ABd^	6.40 ± 0.00^ABe^	6.33 ± 0.15^ABc^	6.47 ± 0.06^Ac^	6.37 ± 0.06^ABef^	6.33 ± 0.12A^Bc^	6.30 ± 0.10^ABd^	6.37 ± 0.06^ABcd^	6.30 ± 0.10^ABd^	6.30 ± 0.10^ABb^
42	6.43 ± 0.06^Af^	6.30 ± 0.00^ABd^	6.63 ± 0.71^ABd^	6.37 ± 0.07^ABc^	6.27 ± 0.07^Bd^	6.30 ± 0.00^ABf^	6.37 ± 0.00^ABc^	6.27 ± 0.07^Bd^	6.27 ± 0.00^Bd^	6.37 ± 0.07^ABcd^	6.40 ± 0.21^ABb^
63	6.60 ± 0.00^Ade^	6.57 ± 0.06^Acd^	6.57 ± 0.06^Ad^	6.40 ± 0.00^Bc^	6.30 ± 0.00^Cd^	6.53 ± 0.06^Ad^	6.37 ± 0.06^BCc^	6.30 ± 0.00^Cd^	6.53 ± 0.06^Ac^	6.40 ± 0.00^Bc^	6.30 ± 0.00^Cb^
84	6.77 ± 0.06^ABcd^	6.80 ± 0.00^Ab^	6.73 ± 0.06^ABCb^	6.67 ± 0.06^BCDb^	6.57 ± 0.06^Db^	6.70 ± 0.00^ABCc^	6.67 ± 0.06^BCDb^	6.63 ± 0.12^CDb^	6.73 ± 0.06^ABCc^	6.70 ± 0.00^ABCb^	6.67 ± 0.12^BCDa^
105	6.93 ± 0.06^Abc^	6.83 ± 0.00^ABCb^	6.87 ± 0.06^ABa^	6.80 ± 0.00^ABCa^	6.70 ± 0.00^Ca^	6.80 ± 0.00^ABCb^	6.80 ± 0.00^ABCa^	6.73 ± 0.06^BCab^	6.90 ± 0.20^Aab^	6.80 ± 0.00^ABCa^	6.73 ± 0.06^BC^a
126	7.00 ± 0.00^Aab^	7.00 ± 0.00^Aa^	6.90 ± 0.10^ABCa^	6.80 ± 0.00^BCDEa^	6.70 ± 0.00^Ea^	6.87 ± 0.12^ABCDab^	6.80 ± 0.00^BCDEa^	6.73 ± 0.06^Dea^b	6.93 ± 0.21^ABab^	6.83 ± 0.06^BCDEa^	6.77 ± 0.06^CDEa^
147	7.13 ± 0.23^Aa^	7.13 ± 0.23^Aa^	6.93 ± 0.06^Ba^	6.87 ± 0.06^Ba^	6.77 ± 0.06^Ba^	6.90 ± 0.00^Ba^	6.83 ± 0.06^Ba^	6.80 ± 0.00^Ba^	6.97 ± 0.06^Aa^	6.87 ± 0.06^Ba^	6.80 ± 0.00^Ba^
TVB-N (mg N/100 g)											
0	2.80 ± 0.00^Ae^	2.80 ± 0.00^Af^	2.80 ± 0.00^Af^	2.80 ± 0.00^Af^	2.80 ± 0.00^Af^	2.80 ± 0.00^Af^	2.80 ± 0.00^Ad^	2.80 ± 0.00^Af^	2.80 ± 0.00^Ae^	2.80 ± 0.00^Af^	2.80 ± 0.00^Af^
21	6.30 ± 0.99^Ade^	6.30 ± 0.99^Ae^	5.60 ± 0.00^ABe^	5.60 ± 0.00A^Be^	4.20 ± 0.00^Be^	6.30 ± 0.99^Ae^	5.60 ± 0.00^ABc^	4.20 ± 0.00^Be^	6.30 ± 0.99^Ade^	6.30 ± 0.99^Ae^	5.60 ± 0.00^ABe^
42	9.80 ± 0.00^Acd^	9.10 ± 0.99^ABd^	7.00 ± 0.00^Dde^	7.00 ± 0.00^Dde^	5.60 ± 0.00^Ed^	7.70 ± 0.99^CDd^	7.00 ± 0.00^Dbc^	5.60 ± 0.00^Ed^	9.80 ± 0.00^Acd^	9.10 ± 0.99^ABd^	7.00 ± 0.00^Dde^
63	10.50 ± 0.99^Acd^	9.80 ± 0.00^ABd^	8.40 ± 0.00^Cd^	7.00 ± 0.00^Dde^	6.30 ± 0.99^Dcd^	8.40 ± 0.00^Cd^	7.00 ± 0.00^Dbc^	7.00 ± 0.00^Dc^	10.50 ± 0.99^Acd^	9.80 ± 0.00^ABd^	8.40 ± 0.00^Cd^
84	13.30 ± 4.95^Abc^	12.60 ± 0.00^Ac^	8.40 ± 0.00^Bd^	8.05 ± 1.48^Bcd^	7.00 ± 0.00^Bc^	9.80 ± 0.00^ABc^	8.40 ± 0.00^Bb^	7.70 ± 0.99^Bc^	13.30 ± 4.95^Abc^	12.60 ± 0.00^Ac^	8.40 ± 0.00^Bd^
105	17.50 ± 0.99^Aab^	16.10 ± 0.99^Ab^	11.20 ± 0.00^CDc^	9.80 ± 0.00^DEbc^	8.40 ± 0.00^Eb^	16.80 ± 0.00^Ab^	12.60 ± 0.00^BCa^	11.20 ± 0.00^CDa^	17.50 ± 0.99^Aab^	16.10 ± 0.99^Ab^	11.20 ± 0.00^CDc^
126	18.20 ± 0.00^Aa^	16.80 ± 0.00^ABb^	14.00 ± 1.98^BCb^	11.20 ± 1.98^CDab^	9.10 ± 0.99^Db^	15.40 ± 0.00^Aba^	11.90 ± 2.97^CDa^	9.80 ± 0.00^Db^	18.20 ± 0.00^Aa^	16.80 ± 0.00^ABb^	14.00 ± 1.98^BCb^
147	20.30 ± 0.99^Aa^	18.20 ± 0.00^Ba^	16.80 ± 0.00^Ba^	12.60 ± 0.00^CDEa^	11.20 ± 0.00^Ea^	16.80 ± 0.00^Ba^	12.60 ± 0.00^CDEa^	11.90 ± 0.99^DEa^	20.30 ± 0.99^Aa^	18.20 ± 0.00^Ba^	16.80 ± 0.00^Ba^

Uppercase letters in the same row indicate statistical differences between groups, lowercase letters in the same column indicate statistical differences within groups throughout storage (*P* < 0.05). C: control without gelatin film, G-C: chicken burgers wrapped with gelatin film, G-G1: burgers wrapped with gelatin film incorporated with 1% grapefruit peel extract, G-G2: burgers wrapped with gelatin film incorporated with 2% grapefruit peel extract, G-G4: burgers wrapped with gelatin film incorporated with 4% grapefruit peel extract, G-O1: burgers wrapped with gelatin film incorporated with 1% orange peel extract, G-O2: burgers wrapped with gelatin film incorporated with 2% orange peel extract, G-O4: burgers wrapped with gelatin film incorporated with 4% orange peel extract, G-L1: burgers wrapped with gelatin film incorporated with 1% lemon peel extract, G-L2: burgers wrapped with gelatin film incorporated with 4% lemon peel extract

### Bioactive components of lemon, orange and grapefruit peel extracts

The peel parts of citrus are rich in phenolic compounds, limonoids and flavonoids (Ramful et al., 2010; Yenice & Akşit, 2026). As shown in Table 2, there was a significant difference in the total antioxidant and phenolic compounds of grapefruit, orange, and lemon peel extracts (*P*<0.05). The peel of grapefruit has the most phenolic compounds and antioxidant activity, followed by the peels of oranges and lemons. This variance may be ascribed to disparities in the content and concentration of phenolic chemicals found in each citrus peel. The antioxidant value of grapefruit peel extract was noted (687.48 µmol trolox/g) in the 70% ethanolic extract, Likewise, lemon peel extract showed the lowest value (227.40 µmol trolox/g). as well, according to the results, phenolic compounds were noted in the grapefruit (598.45 mg GAE/g), orange peel (428.62 mg GAE/g), and lemon peel (364.8 mg GAE/g), respectively. The existing outcomes are in line with study of Ramful et al. (2010) who reported that citrus peels changed from 188.2 to 766.7 mg GAE/100 g in total phenolics. Similarity, Bağdatlı and Khalily (2022) highlighted that 70% ethanolic orange peel extract has strong antioxidant activity with the highest value as 607.67 µmol trolox/g, then lemon peel extract 203.22 µmol trolox/g. as well, Ashraf et al. (2024) observed that total phenolics (189.7 mg GAE/100 g) were noted in 50% ethanolic orange peel extract (OPE). The findings agree with the study Bağdatlı and Khalily (2022) who noted that the total antioxidant activity and phenolic content of grapefruit peel are higher than those of mandarin peel.

**Table 2:** Total phenolic compounds and antioxidant capacity of grapefruit, orange, and lemon peel extracts

Samples	Total phenolic compounds Mg gae/g	Total antioxidant capacity (µmol trolox/g)
Grapefruit peel	598.45 ± 112.16^a^	687.48 ± 94.60^a^
Orange peel	428.62 ± 105.82^a^	533.91 ± 102.80^ab^
Lemon peel	364.8 ± 64.61^ab^	227.40 ± 170.36^b^

Different small letters indicate a significant difference among samples (*P* < 0.05). Values are expressed as mean ± standard deviation (*n* = 3)

Peroxides are the primary reactive products of lipid oxidation and are responsible for initial oxidation (Park & Kim, 2016). At the beginning of the storage, the PV value was noted as 1.00 meq O₂/kg, and that value increased in all samples during storage (Table 3). Samples with gelatin films wrapping citrus peel extract (groups G-G, G-O, and G-L) maintained significantly lower PV values throughout storage (*P* < 0.05). At the end of storage, the PV value of the control was noted as 7.00 meq O₂/kg, and G-G4 was noted as 4.50 meq O₂/kg. These findings align with previous reports. Panahi et al. (2022) reported that the increase in PV value over time is attributed to the increase in oxidation with storage time. Similarly, Azizkhani et al. (2023) observed that incorporating bitter orange peel extract into various coatings for chicken fillets yielded the lowest PV during cold storage.

A similar trend was observed in TBARS values, a secondary indicator of lipid oxidation indicating odor alterations, color, and off-flavor. The initial TBARS level was 0.15 mg MDA/kg in treated and untreated chicken burgers (Table 3). Furthermore, by increasing the storage time, the TBARS values of all tested burgers were increased (*p* < 0.05). At the 147th day of storage, the TBARS values were in groups C and G-C (1.01 and 0.92 mg MDA/kg) and in citrus peel extract film groups: G-G4 (0.74 mg MDA/kg), G-O4 (0.75 mg MDA/kg), and G-L4 (0.80 mg MDA/kg). These results are consistent with those of Ebrahimian et al. (2023) who found that treated samples (gelatin with orange peel extract) showed the highest antioxidant activity during storage. Panahi et al. (2022) also reported that the TBARS value of chicken that was treated with edible films combined with citrus extract was almost lower than that of the control sample.

As illustrated in Table 3, the peroxide value (PV) and thiobarbituric acid reactive substances (TBARS) values of all samples increased gradually during storage, suggesting that lipid oxidation occurred in chicken burgers. Samples covered with gelatin films containing citrus peel extracts exhibited significantly lower peroxide value (PV) and thiobarbituric acid reactive substances (TBARS) values than the control group throughout frozen storage. The reduced lipid oxidation may be attributed to the presence of naturally occurring phenolic compounds, flavonoids (e.g., hesperidin and naringin), and other antioxidant phytochemicals in citrus peel extracts, which effectively scavenge free radicals, donate hydrogen atoms, and inhibit oxidative chain reactions in lipid-rich food systems (Yun & Liu, 2022). Furthermore, increasing the concentration of citrus peel extracts incorporated into the gelatin films significantly enhanced their ability to retard lipid oxidation, indicating a concentration-dependent protective effect. Similar findings have been reported in previous studies, demonstrating that edible films enriched with plant-derived antioxidants effectively improve the oxidative stability and shelf life of meat products during storage (Farokhzad et al., 2023; Moura-Alves et al., 2023; Jridi et al., 2019). These findings indicate that gelatin edible films enriched with citrus peel extracts can effectively preserve the oxidative stability of chicken burgers and delay lipid deterioration during frozen storage.

**Table 3:** Changes in peroxide value (PV) and thiobarbituric acid reactive substances (TBARS) of chicken burgers coated with gelatin films enriched with grapefruit, orange, and lemon peel extracts during frozen storage (− 18 °C) for 147 days

Storage (days)	C	C-G	G-G1	G-G2	G-G4	G-O1	G-O2	G-O4	G-L1	G-L2	G-L4
PV (meq O_2_/kg)											
0	1.00 ± 0.00^Af^	1.00 ± 0.00^Ad^	1.00 ± 0.00^Af^	1.00 ± 0.00^Ae^	1.00 ± 0.00^Ac^	1.00 ± 0.00^Ac^	1.00 ± 0.00^Ad^	1.00 ± 0.00^Ac^	1.00 ± 0.00^Ae^	1.00 ± 0.00^Ae^	1.00 ± 0.00^Ad^
21	1.50 ± 0.71^Ae^f	1.00 ± 0.00^Ad^	1.50 ± 0.71^Aef^	1.00 ± 0.00^Ae^	0.50 ± 0.71^Ac^	1.00 ± 0.00^Ac^	1.00 ± 0.00^Ad^	0.50 ± 0.71^Ac^	1.00 ± 0.00^Ae^	1.00 ± 0.00^Ae^	1.00 ± 0.00^Ad^
42	2.00 ± 0.00^Ae^	2.00 ± 0.00^Ad^	2.00 ± 0.00^Aef^	1.50 ± 0.71^Ade^	1.00 ± 0.00^Ac^	2.50 ± 0.71^Ab^	1.50 ± 0.71^Ad^	1.00 ± 0.00^Ac^	3.00 ± 0.00^Ad^	2.00 ± 1.41^Ade^	1.50 ± 2.12^Acd^
63	3.00 ± 0.00^AB^d	3.50 ± 0.71^Ac^	2.50 ± 0.71^ABde^	2.00 ± 0.00^Bd^	1.50 ± 0.71^Bbc^	3.00 ± 0.00^ABb^	3.00 ± 0.00^ABc^	1.50 ± 0.71^Bc^	3.50 ± 0.71^Acd^	3.00 ± 1.41^ABcd^	2.50 ± 0.71^ABbcd^
84	4.50 ± 0.71^Ac^	4.00 ± 0.00^ABc^	3.50 ± 0.71^ABcd^	3.00 ± 0.00^Bc^	2.00 ± 0.00^Cbc^	3.50 ± 0.71^ABb^	4.00 ± 0.00^ABb^	3.00 ± 0.00^Bb^	4.00 ± 0.00^AB^c	4.00 ± 0.00^ABbc^	3.50 ± 0.71^Ababc^
105	5.00 ± 0.00^Ac^	5.00 ± 0.00^Ab^	4.50 ± 0.00^ABbc^	4.00 ± 0.00^Bb^	3.00 ± 0.00^Cab^	5.00 ± 0.00^Aa^	4.50 ± 0.71^ABab^	4.00 ± 0.00^Ba^	5.00 ± 00^Ab^	4.50 ± 0.71^Ababc^	4.00 ± 0.00^Bab^
126	6.00 ± 0.00^Ab^	5.50 ± 0.71^ABb^	5.00 ± 0.00^ABCab^	5.00 ± 0.00^ABCa^	4.00 ± 0.00^Ca^	5.50 ± 0.71^ABa^	5.00 ± 0.00^ABCab^	4.50 ± 0.71^BCa^	6.00 ± 0.00^Aa^	5.00 ± 0.00^ABCab^	4.50 ± 0.71^BCab^
147	7.00 ± 0.00^Aa^	6.50 ± 0.71^ABb^	6.00 ± 0.00^ABCa^	5.50 ± 0.71^BCDa^	4.50 ± 0.00^Da^	6.00 ± 0.00A^BCa^	5.50 ± 0.71^BCDa^	5.00 ± 0.00^CDa^	6.50 ± 0.71^Aba^	6.00 ± 0.00^ABCa^	5.50 ± 0.71^BCDa^
TBARS (mg MDA/kg)											
0	0.15 ± 0.01^Ag^	0.15 ± 0.01^Ae^	0.15 ± 0.01^Ae^	0.15 ± 0.01^Ae^	0.15 ± 0.01^Ae^	0.15 ± 0.01^Ad^	0.15 ± 0.01^Ad^	0.15 ± 0.01^Ae^	0.15 ± 0.01^Ag^	0.15 ± 0.01^Af^	0.15 ± 0.01^Ae^
21	0.26 ± 0.04^Af^	0.20 ± 0.01^Be^	0.18 ± 0.01^CDe^	0.18 ± 0.01^CDde^	0.17 ± 0.01^De^	0.18 ± 0.00^CDd^	0.18 ± 0.00^CDd^	0.17 ± 0.09^De^	0.19 ± 0.01^CDf^	0.18 ± 0.01^CDf^	0.17 ± 0.01^Dde^
42	0.40 ± 0.02^Ae^	0.37 ± 0.04^Bd^	0.28 ± 0.03^Dd^	0.23 ± 0.03^Ecd^	0.19 ± 0.07^Fde^	0.33 ± 0.02^Cc^	0.29 ± 0.05^Dc^	0.22 ± 0.12^Ed^	0.30 ± 0.04^De^	0.24 ± 0.02^Ee^	0.22 ± 0.07^Ed^
63	0.51 ± 0.03^Ad^	0.46 ± 0.06^Bc^	0.34 ± 0.05^DEc^	0.26 ± 0.03^FGbc^	0.21 ± 0.12^Hcd^	0.36 ± 0.4^Dc^	0.30 ± 0.03^EFc^	0.24 ± 0.14^GHd^	0.41 ± 0.03^Cd^	0.34 ± 0.03^Dd^	0.36 ± 0.02^Dc^
84	0.67 ± 0.01^Ac^	0.62 ± 0.03^Bb^	0.41 ± 0.00^DEb^	0.30 ± 0.05^Gb^	0.22 ± 0.02^Hc^	0.43 ± 0.01^Db^	0.34 ± 0.00^FGc^	0.36 ± 0.00^EFc^	0.51 ± 0.00^Cc^	0.43 ± 0.03^Dc^	0.41 ± 0.01^DEc^
105	0.96 ± 0.00^Ab^	0.89 ± 0.06^Aa^	0.87 ± 0.03^ABa^	0.78 ± 0.67^BCa^	0.67 ± 0.02^Db^	0.90 ± 0.00^Aa^	0.75 ± 0.06^CDb^	0.68 ± 0.00^Db^	0.92 ± 0.02^Ab^	0.87 ± 0.08^Ab^	0.71 ± 0.00^CDb^
126	0.97 ± 0.00^Ab^	0.91 ± 0.06^ABa^	0.88 ± 0.02^BCa^	0.81 ± 0.01^Dea^	0.70 ± 0.01^Fb^	0.89 ± 0.03^BCa^	0.82 ± 0.00^DEa^	0.73 ± 0.01^Fa^	0.92 ± 0.02^Aba^	0.85 ± 0.02^CDa^	0.78 ± 0.01^Ea^
147	1.01 ± 0.02^Aa^	0.92 ± 0.02^BCa^	0.89 ± 0.00^BCDa^	0.83 ± 0.01^Dea^	0.74 ± 0.03^Fa^	0.86 ± 0.04^BCDEa^	0.83 ± 0.01^DEa^	0.75 ± 0.03^Fa^	0.93 ± 0.04^Ba^	0.86 ± 0.01^CDEa^	0.80 ± 0.06^EFa^

Statistical differences between groups and days (*P* < 0.05). C: control without gelatin film, G-C: chicken burgers wrapped with gelatin film, G-G1: burgers wrapped with gelatin film incorporated with 1% grapefruit peel extract, G-G2: burgers wrapped with gelatin film incorporated with 2% grapefruit peel extract, G-G4: burgers wrapped with gelatin film incorporated with 4% grapefruit peel extract, G-O1: burgers wrapped with gelatin film incorporated with 1% orange peel extract, G-O2: burgers wrapped with gelatin film incorporated with 2% orange peel extract, G-O4: burgers wrapped with gelatin film incorporated with 4% orange peel extract, G-L1: burgers wrapped with gelatin film incorporated with 1% lemon peel extract, G-L2: burgers wrapped with gelatin film incorporated with 2% lemon peel extract, G-L4: burgers wrapped with gelatin film incorporated with 4% lemon peel extract

### Microbiological changes

**Fig. 1:**
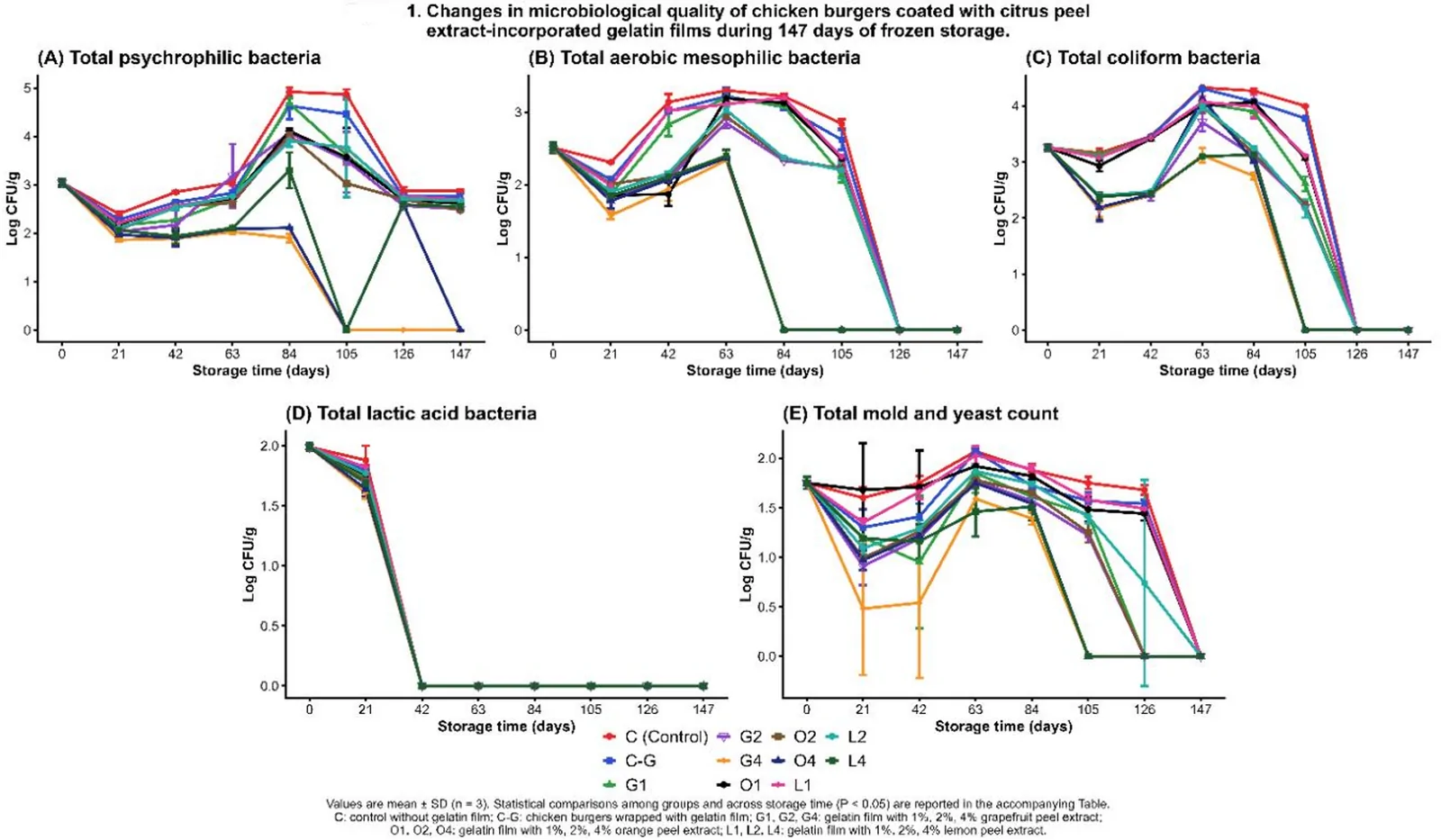
Changes in the microbiological quality of chicken burgers coated with citrus peel extract-incorporated gelatin films during 147 days of frozen storage: (**A**) total psychrophilic bacteria, (**B**) total aerobic mesophilic bacteria, (**C**) total coliform bacteria, (**D**) total lactic acid bacteria, and (**E**) total mold and yeast count. Values are expressed as mean ± SD (*n* = 3). Microbial counts are expressed as log CFU/g. Different letters in the corresponding data table indicate significant differences among treatments and storage periods (*P* < 0.05)

As illustrated in Fig. 1; Table 4, chicken burgers coated with citrus peel extract-incorporated gelatin films consistently exhibited lower microbial counts than the control throughout the frozen storage period. Because all samples were stored at − 18 °C, active microbial proliferation is expected to be minimal under frozen conditions. Therefore, the lower microbial counts observed in the treated samples should be interpreted as reflecting the intrinsic antimicrobial activity of the citrus peel extracts and their contribution to maintaining improved microbiological quality during storage rather than inhibition of active microbial growth. The antimicrobial activity of the citrus peel extract-incorporated gelatin films may be attributed to the presence of naturally occurring phenolic compounds, flavonoids (e.g., hesperidin and naringin), essential oils, and other phytochemicals present in citrus peels. These bioactive compounds have been reported to disrupt microbial cell membrane integrity, increase membrane permeability, interfere with essential enzymatic activities, and consequently reduce the survival and persistence of spoilage microorganisms (Yun & Liu, 2022; Jridi et al., 2019; Ucak et al., 2021). Furthermore, increasing the concentration of citrus peel extracts from 1% to 2% and 4% enhanced the antimicrobial effectiveness of the gelatin films, indicating a concentration-dependent preservative effect. Similar findings have been reported for edible films enriched with plant-derived antioxidants and citrus peel extracts, which effectively improve the microbiological quality and shelf life of meat products during storage (Moura-Alves et al., 2023; Ucak et al., 2021). In the microbial analysis, fluctuations between groups and days were seen. This fluctuation may be related to several factors that contributed to this phenomenon, including the effects of biopolymers, natural additives, and thermal shock (Capozzi et al., 2011). Overall, these findings demonstrate that gelatin edible films enriched with citrus peel extracts possess intrinsic antimicrobial activity that contributes to reducing microbial load and maintaining the microbiological quality of chicken burgers during frozen storage.

Total psychrotrophic bacteria play a significant role in the spoilage of chill-stored meat (Pothakos et al., 2014). Significant differences (*P* < 0.05) were observed between the control and treatment groups throughout the storage period. At the beginning of storage TPC was noted (3.03 log CFU/g) as shown in Fig. 1. In the comparison of average values be-tween groups, the control group showed the highest total psychrophilic count (3.36 log CFU/g). The lowest total psychrophilic count (1.34 log CFU/g) was observed in the G-G4 group as shown in Table 4. These findings agree with those of (Abdel-Naeem et al., 2022), who reported that the bacterial counts were lower in treated groups with lemon, orange, and grapefruit peel powder than in the control, possibly due to their phenolic acid content. In this study, none of the groups, including the control, exceeded the acceptable limit until the last day of analysis.

Similarly, the total aerobic mesophilic count (TMB) is an important indicator for assessing the microbiological quality and safety of meat and meat products during storage, whether fresh or frozen. As shown in Fig. 1, the comparison of mean values between the treatment groups revealed statistically significant differences (*p* < 0.05) in total aerobic mesophilic counts. The highest TMB values were observed in the control and G-C groups (2.17 and 2.06 log CFU/g, respectively), and the lowest values (1.05 and 1.09 log CFU/g) were recorded in both the G-G4 and G-O4 groups, as shown in Table 4. Ibrahim et al. (2018) reported that incorporating orange or lemon peel powders into beef patties significantly reduced aerobic plate counts (APC) during chilled storage. Similarly, Alahakoon et al. (2013) reported that citrus peel extracts and calcium lactate marinades inhibited the growth of total aerobic bacteria on chicken breast during storage. In this study, the positive action of gelatin films with citrus peels at a 4% concentration was noted on the total aerobic mesophilic count. Table 4 summarizes the mean microbiological counts obtained from all sampling intervals throughout the 147-day frozen storage period for each treatment group.

**Table 4:** Mean microbiological counts (log CFU/g) of chicken burgers coated with citrus peel extract-incorporated gelatin films during the 147-day frozen storage period

Groups	Total psychrophilic bacteria	Total aerobic mesophilic count	Total coliform counts	Total lactic acid bacteria	Total mold and yeast count
log CFU/g					
C	3.36 ± 0.97^a^	2.17 ± 1.3^8a^	2.81 ± 1.79^a^	1.93 ± 0.08^a^	1.56 ± 0.15^a^
C-G	3.18 ± 0.87^ab^	2.06 ± 1.32^a^	2.75 ± 1.74^a^	1.89 ± 0.14^a^	1.42 ± 0.25^a^
G-G1	2.97 ± 0.83^ab^	1.95 ± 1.29^a^	2.54 ± 1.63^a^	1.86 ± 0.18^a^	1.10 ± 0.64^a^
G-G2	2.88 ± 0.69^ab^	1.73 ± 1.11a	2.11 ± 1.41^a^	1.84 ± 0.21^a^	1.05 ± 0.62^a^
G-G4	1.34 ± 1.17^c^	1.05 ± 1.15^ab^	1.71 ± 1.46^b^	1.80 ± 0.26^a^	0.72 ± 0.74^ab^
G-O1	2.94 ± 0.62^ab^	1.87 ± 1.25^a^	2.59 ± 1.65^a^	1.88 ± 0.16^a^	1.48 ± 0.17^a^
G-O2	2.82 ± 0.5^ab^	1.77 ± 1.13^a^	2.18 ± 1.46^a^	1.85 ± 0.19^a^	1.09 ± 0.62^a^
G-O4	1.71 ± 1.12^c^	1.09 ± 1.19^ab^	1.88 ± 1.66^ab^	1.81 ± 0.25^a^	0.90 ± 0.76^ab^
G-L1	2.96 ± 0.59^ab^	2.03 ± 1.32^a^	2.62 ± 1.66^a^	1.90 ± 0.12^a^	1.47 ± 0.23^a^
G-L2	2.94 ± 0.62^ab^	1.77 ± 1.14^a^	2.19 ± 1.48^a^	1.86 ± 0.19^a^	1.24 ± 0.41^a^
G-L4	2.20 ± 1.01^bc^	1.11 ± 1.20^ab^	1.79 ± 1.51^ab^	1.84 ± 0.21^a^	0.91 ± 0.74^ab^

The comparison of average value between groups, Lowercase letters indicate statistical differences between groups (*P* < 0.05)

Table 4 illustrates that the counts of total psychrophilic bacteria (TPB), total aerobic mesophilic bacteria (TMB), total coliform bacteria (TCB), lactic acid bacteria (LAB), and total mold and yeast increased gradually throughout the storage period in all samples. As summarized in Table 4, chicken burgers coated with citrus peel extract-incorporated gelatin films exhibited lower mean microbial counts than the control throughout the frozen storage period. The greatest reduction was observed in films containing 4% grapefruit peel extract, indicating a concentration-dependent antimicrobial effect. When compared to the treatment groups, the control samples showed much higher microbial counts. Conversely, chicken burgers that were coated with gelatin films that were enhanced with grapefruit, orange, and lemon peel extracts exhibited significantly reduced microbial proliferation. The antibacterial action is due to phenolic chemicals and other bioactive ingredients in citrus peel extracts that suppress microbial development. Additionally, the antibacterial efficacy of the gelatin films was increased by increasing the content of citrus peel extracts (1%, 2%, and 4%), which improved the microbiological stability of the chicken burgers during storage.

Total coliform counts followed a similar trend. The data in Fig. 1 shows the overall number of coliform bacteria present in both untreated (control) and treated samples. The findings indicate that initially, there were 2.1 CFU/g of bacteria. On the Table 4, the comparison of average values between groups throughout the storage period showed that the control groups (C and G-C) exhibited the highest TCB values, at 2.81 and 2.75 log CFU/g, respectively. In contrast, samples treated with gelatin-based coatings incorporating citrus peel extracts (groups G-G4, G-O4, and G-L4) showed inhibition of coliform bacteria. Among all treatments, gelatin films containing 4% grapefruit peel extract (G-G4) exhibited the lowest mean counts of psychrophilic bacteria, total aerobic mesophilic bacteria, coliforms, lactic acid bacteria, and molds and yeasts, indicating the strongest antimicrobial effectiveness among the tested formulations. The present findings agreed with those reported by Arsène et al. (2021) who indicated that grapefruit peels, as an agricultural byproduct, possess notable an-tibacterial properties that may help combat antibiotic resistance and support the de-velopment of new treatments for bacterial diseases.

The total lactic acid bacteria (LAB) demonstrated no significant differences between the control and treated groups throughout storage, up to 147 days, as shown in Fig. 1. After 42 days there were not any lactic acid colonies until the last day of storage. As Capozzi et al. (2011) reported that one of the main factors in the destruction of lactic acid bacteria is cold stress. Although the mean values of the groups were different in the control, G-L4, G-O4 and G-G4 groups (1.93, 1.84, 1.81 and 1.80 log CFU/g, respec-tively) on the Table 4. Moustafa et al. (2022) reported that incorporating citrus powders into minced beef significantly reduced the microbial load compared to untreated controls. Similarly, Ebrahimian et al. (2023) suggested that the phenolic compounds present in citrus peel extracts may inhibit bacterial growth and enhance the resistance of chicken meat to endogenous enzymatic activity.

All the treatment burgers covered with citrus peel-gelatin-based edible films containings of grapefruit (4, 2, 1%), orange (4, 2, 1%), and lemon (4, 2, 1%) examined in the present study caused significantly lower (*P* ≤ 0.05) counts for yeast and mold in chicken burgers as compared to the control samples during storage. At the beginning of the storage, total yeast and mold was 1.75 log CFU/g Fig. 1. The comparison of average values between groups throughout storage showed that the treated groups consistently had lower microbial counts than the controls. The highest total mold and yeast count was found in the control group (1.56 log CFU/g), while the lowest values (0.72, 0.90, and 0.91 log CFU/g) were recorded in the G-G4, G-O4, and G-L4 groups, respectively. As with the previous investigations, Petrou et al. (2022) and Vlaicu et al. (2020) reported that citrus peels and oils inhibited the growth of yeast and mold, pathogenic *Escherichia coli*, and *Staphylococcus* spp. Similarly, Ukom et al. (2022) found that grapefruit peel powder (GFPP) effectively inhibited yeast and mold growth.

### Bioactive components of lemon, orange and grapefruit peel extracts

The peel parts of citrus are rich in phenolic compounds, limonoids and flavonoids (Ramful et al., 2010). As shown in Table 2, there was a significant difference in the total antioxidant and phenolic compounds of grapefruit, orange, and lemon peel extracts (*P*<0.05). The peel of grapefruit has the most phenolic compounds and antioxidant activity, followed by the peels of oranges and lemons. This variance may be ascribed to disparities in the content and concentration of phenolic chemicals found in each citrus peel. The antioxidant value of grapefruit peel extract was noted (687.48 µmol trolox/g) in the 70% ethanolic extract, Likewise, lemon peel extract showed the lowest value (227.40 µmol trolox/g). as well, according to the results, phenolic compounds were noted in the grapefruit (598.45 mg GAE/g), orange peel (428.62 mg GAE/g), and lemon peel (364.8 mg GAE/g), respectively. The existing outcomes are in line with study of Ramful et al. (2010) who reported that citrus peels changed from 188.2 to 766.7 mg GAE/100 g in total phenolics. Similarity, Bağdatlı and Khalily (2022) highlighted that 70% ethanolic orange peel extract has strong antioxidant activity with the highest value as 607.67 µmol trolox/g, then lemon peel extract 203.22 µmol trolox/g. as well, Ashraf et al. (2024) observed that total phenolics (189.7 mg GAE/100 g) were noted in 50% ethanolic orange peel extract (OPE). The findings agree with the study Bağdatlı and Khalily (2022) who noted that the total antioxidant activity and phenolic content of grapefruit peel are higher than those of mandarin peel.

The superior preservative performance of grapefruit peel extract observed in the present study is likely attributable to differences in its phytochemical composition rather than total phenolic content alone. Grapefruit peel is particularly rich in the flavanone naringin, together with furanocoumarins, limonoids, phenolic acids, and essential oil constituents, whereas orange peel contains higher concentrations of hesperidin and lemon peel is characterized by abundant eriocitrin and other flavanone glycosides (Goulas & Manganaris, 2012; Munir et al., 2024). These phytochemicals possess complementary antioxidant and antimicrobial properties through multiple mechanisms, including free radical scavenging, metal ion chelation, inhibition of lipid peroxidation, disruption of microbial cell membranes, and interference with microbial metabolic processes (Moura-Alves et al., 2023; Jridi et al., 2019). Consequently, the combined action and relative abundance of these bioactive constituents may explain the superior antioxidant and antimicrobial effectiveness of grapefruit peel extract observed in the present study. Nevertheless, comprehensive phytochemical characterization of the citrus peel extracts using analytical techniques such as HPLC or GC-MS would provide a more detailed understanding of the compounds responsible for these effects and should be considered in future studies.

Although the present study demonstrated the potential of citrus peel extract-incorporated gelatin edible films to improve the physicochemical stability and microbiological quality of chicken burgers during frozen storage, several limitations should be acknowledged. First, the antioxidant properties of the composite gelatin films after incorporation of citrus peel extracts were not directly evaluated, although the antioxidant activity of the individual citrus peel extracts was determined. Because incorporation into the gelatin matrix may influence the functional properties of the bioactive compounds, future studies should investigate the antioxidant characteristics of the developed edible films. Second, the pH of the citrus peel extracts and the prepared gelatin films was not measured, which may provide additional insight into their physicochemical behavior and preservative mechanisms. Furthermore, while the present study demonstrated the preservative effectiveness of citrus peel extract-incorporated gelatin films under frozen storage conditions, additional investigations under refrigerated storage and with more detailed characterization of the underlying antioxidant and antimicrobial mechanisms would further improve understanding of their functionality. Finally, comprehensive characterization of the bioactive compounds released from the edible films during storage would provide valuable information regarding their contribution to the preservation of meat products and should be considered in future research. Future studies should also evaluate the scalability, mechanical properties, consumer sensory acceptance, and commercial applicability of citrus peel extract-incorporated gelatin edible films to facilitate their potential use as sustainable active packaging materials in the food industry.

## Conclusions

The present study demonstrated that gelatin-based edible films enriched with grapefruit, orange, and lemon peel extracts have considerable potential as sustainable active packaging materials for preserving frozen chicken burgers. Incorporation of citrus peel extracts improved the antioxidant and antimicrobial performance of the gelatin films, with grapefruit peel extract exhibiting the greatest preservative effectiveness among the tested treatments. Chicken burgers coated with citrus peel extract-incorporated gelatin films consistently exhibited lower pH, total volatile basic nitrogen (TVB-N), peroxide value (PV), thiobarbituric acid reactive substances (TBARS), and microbial counts than the uncoated control throughout frozen storage, indicating improved physicochemical stability and extended shelf life. The preservative effects increased with extract concentration, with the 4% formulations providing the greatest protection. Overall, these findings demonstrate that citrus processing by-products can be successfully valorized as natural preservatives in biodegradable gelatin-based edible films, providing an environmentally sustainable approach for improving meat preservation while supporting the development of active food packaging systems. Future studies should further investigate the physicochemical characteristics of the developed films, elucidate the mechanisms underlying their antioxidant and antimicrobial activities, evaluate their performance under different storage conditions, and assess their commercial applicability in food packaging.

## Data Availability

No datasets were generated or analysed during the current study.
